# Effects of geo‐climate factors on phenotypic variation in cone and seed traits of *Pinus yunnanensis*

**DOI:** 10.1002/ece3.10568

**Published:** 2023-09-28

**Authors:** Chengjie Gao, Fangyan Liu, Yingchun Miao, Jin Li, Zirui Liu, Kai Cui

**Affiliations:** ^1^ State Key Laboratory of Tree Genetics and Breeding, Institute of Highland Forest Science Chinese Academy of Forestry Kunming China

**Keywords:** climate factors, intraspecific variation, *Pinus*, population development, reproductive traits

## Abstract

Evaluating variations in reproductive traits and the response of the variations to geo‐climate conditions are essential for understanding the persistence, evolution, and range dynamics of plant populations. However, there are insufficient studies to attempt to analyze the importance of geo‐climate factors in explaining within‐ or among‐population variation in reproductive traits. We examined 14 traits for 2671 cones of *Pinus yunnanensis* collected from nine populations in the mountains of Southwest China to characterize the patterns of phenotypic variation of traits and estimate environmental effects on these trait performances and trait variation. We found the contribution of intrapopulation variation to the overall variation was greater than the interpopulation variation and the larger coefficients of variation for the populations lying at the edge of northern and southern regions. Climatic variables are more important than geographical and tree size variables in their relationships to cone and seed traits. Populations in more humid and warmer climate expressed greater cone and seed weight and seed number but lower seed abortion rate, while the larger coefficients of variation in seed weight and number were detected in northern and southern marginal regions with drier or colder climate. Our study illustrates that intraspecific trait variation should be considered when examining plant species response to changing climate and suggests that the high variability rather than high quality of seed traits in the marginal regions with drier or colder climate might foster plant‐population persistence in stressful conditions.

## Graphical abstract

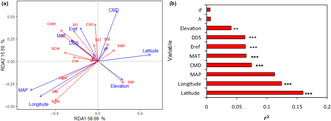

## 1 INTRODUCTION

Understanding species response to climate change including current global warming and altered precipitation regimes is of fundamental importance for assessing their evolutionary and ecological dynamics and for predicting population development and conservation (Cochrane et al., 2015; Fréjaville et al., 2020; Murray et al., 2004; Roland et al., 2014). Species may respond to climate change by locally adapting themselves to the new conditions via phenotypic variation (Cochrane et al., 2016; Henn et al., 2018; Wu et al., 2018). This progress has been mostly built on approaches using mean trait values per species, without considering trait variability within species, relying on the assumption that intraspecific trait variabilities could be neglected relative to interspecific variabilities (Fricke et al., 2019; Soper‐Gorden et al., 2016; Wang et al., 2014). However, it is generally assumed that the species level, even within populations, captures a major part of trait variation (Fricke et al., 2019; Wyse & Hulme, 2021). Intraspecific trait variation arises from a combination of genetic variation, developmental instability (i.e., the inability of an individual to produce a specific phenotype in given environmental conditions), and phenotypic plasticity owing to environmental change across time, including their interaction (Henn et al., 2018; Lemke et al., 2015) and is affected by abiotic and biotic factors such as climate and species interactions (Cochrane et al., 2015; Wu et al., 2018). Since phenotypic traits and their intraspecific variability are closely linked responses to environmental change and provide the basis for natural selection and evolution (Liu et al., 2019; Wyse & Hulme, 2021), studying patterns of phenotypic variation within species along broad ecological and climatic gradients is a powerful framework to understand the patterns and processes that govern phenotypic expression and variation (Leal‐Sáenz et al., 2020). In many cases, gradual trends were evident where among‐population differences in phenotypic traits correlated with latitude, elevation or precipitation and temperature (Cochrane et al., 2016; Mamo et al., 2006; Murray et al., 2004; Wang et al., 2014; Wu et al., 2018). Such studies, however, only provide insight into the average response of phenotypic expression to large‐scale environmental conditions. Less attention has been given to the question on how trait variation within populations differs between regions. Recently, research has started to focus on trait variation within population, and there is a growing awareness of the importance of a general understanding of trait variation within population and its underlying causes across ecologically relevant spatial and temporal scales (Helsen et al., 2017; Henn et al., 2018; Kuppler et al., 2020; Lemke et al., 2015).

Reproductive traits are important phenotypic traits that have been identified as key fitness‐related traits, influencing germination, recruitment, seedling size and growth, tolerance of stress and the probability of survival, thereby determining the natural development and distribution of plant species (Cochrane et al., 2015). For example, larger seeds confer advantages to the plant during early seedling establishment, producing larger seedlings that appear to be more drought resistant, whereas small seeds are thought to have an advantage associated with dispersal to many sites where they may experience favorable abiotic conditions or little competition, but have lower ability to tolerate stressful abiotic conditions or plant competitors (Saatkamp et al., 2019). Alternatively, the number of seeds and seed abortion are comprehensive reflections of many factors such as mating system, pollination efficiency and environment, which can directly contribute to its colonization ability (Mazer et al., 2020). Additionally, cone traits, such as cone size, scale size, and number, may relate to abiotic conditions and resource availability, which are important indicators of reproductive fitness (Mao et al., 2009; Wyse & Hulme, 2021). Population or provenance variation with respect to cone and seed traits is well documented for a number of tree species (Ji et al., 2011; Mamo et al., 2006; Singh & Thapliyal, 2012; Zhang et al., 2022). These studies have revealed the differences existed in cone and seed characters among and within populations. Quantifying reproductive variation within a widely distributed species across broad geographical and climatic gradients is often necessary to identify the relative importance of different environmental factors in relation to variation in intraspecific reproductive traits (Ji et al., 2011; Leal‐Sáenz et al., 2020; Middleton et al., 2021). Several studies have reported trait variation at various geographical scales in forest trees (Gárate‐Escamilla et al., 2019; Sork et al., 2013; Wang et al., 2013), generally following climatic gradients. Seed traits such as length, width, and mass of seeds are strongly influenced by geographical origins (Liu et al., 2013; Loewe‐Muñoz et al., 2020; Mamo et al., 2006). A clear trend of increasing seed mass as latitude decreases from both poles toward the equator has been reported within species in some studies (Leal‐Sáenz et al., 2020; Middleton et al., 2021; Moles et al., 2007; Murray et al., 2004). These geographical trends of seed traits are mainly or partly driven by climatic variables, such as temperature and precipitation (Soper‐Gorden et al., 2016). For example, mean annual temperature has been found to be positively associated with mean seed mass (Murray et al., 2004). In some species, larger seeds are associated with warmer or drier climates (Liu et al., 2013), whereas in others, smaller seeds are associated with warmer or drier conditions (Cochrane et al., 2016). Therefore, it is essential to consider a range of climatic conditions when examining the phenotypic variation within and between populations to understand the patterns and processes that govern phenotypic expression and variation. The species studied thus far differ dramatically in range size and in the steepness of the climatic gradients and environmental heterogeneity experienced across their ranges; however, there are insufficient studies to attempt to analyze the importance of these factors in explaining among‐population variation in reproductive traits (Cochrane et al., 2015), and less attention has been given to the variation of reproductive traits within populations and about differences in this variation on geo‐climate gradients. Previous studies with respect to phenotypes variation in reproductive traits within species usually focused on annual, perennial herb (Helsen et al., 2017; Murray et al., 2004; Soper‐Gorden et al., 2016) or invasive species (Liu et al., 2019), with little attention paid to woody plants species (Wang et al., 2014).

Conifers are an especially promising species in which to investigate variation in reproductive traits because they have the widest geographic distribution, and are ecologically diverse (Liu & El‐Kassaby, 2020). *Pinus yunnanensis* Franch. is a typical endemic coniferous species that constitutes the principal subtropical coniferous forests in the mountains of Southwest China region (Jin & Peng, 2004). It has been demonstrated that there is high level of genetic diversity in *P. yunnanensis*, and the morphological characteristics show significantly different patterns of variation in different eco‐geographical backgrounds. This relatively high level of the existence of ecotypes adapted to different climatic conditions (Wang et al., 2013) provides the foundation for studying the intraspecific variation in reproductive traits at a regional scale. Given the ecological importance of *P. yunnanensis* and the multiple challenges to future regeneration of the species, it is critical to understand the patterns of variation in reproductive structures and factors underlying the phenotypic trait variation in order to provide valuable information for species' management and conservation in the future. Here, we measured 14 cone and seed traits for 2671 cones of *P. yunnanensis* collected from nine populations in the mountains of Southwest China. We examined the patterns of phenotypic variation of cone and seed traits among and within population and analyzed the relationships between environmental factors (climate, geography, and tree size) and phenotypic traits. We focused on the relative importance of the effects of environmental factors on these reproductive traits. In addition, we explored the effects of geo‐climate variables on the trait variation within populations. Specifically, we aimed to explore the following questions: (i) Are there substantial variations of reproductive traits among and within populations? If so, do phenotypic traits or variation vary significantly along geo‐climate gradients? (ii) What is the relative importance of environmental factors (climate, geography, and tree size) in affecting reproductive trait variations?

## 2 MATERIALS AND METHODS

### 2.1 Species and study sites

**FIGURE 1:**
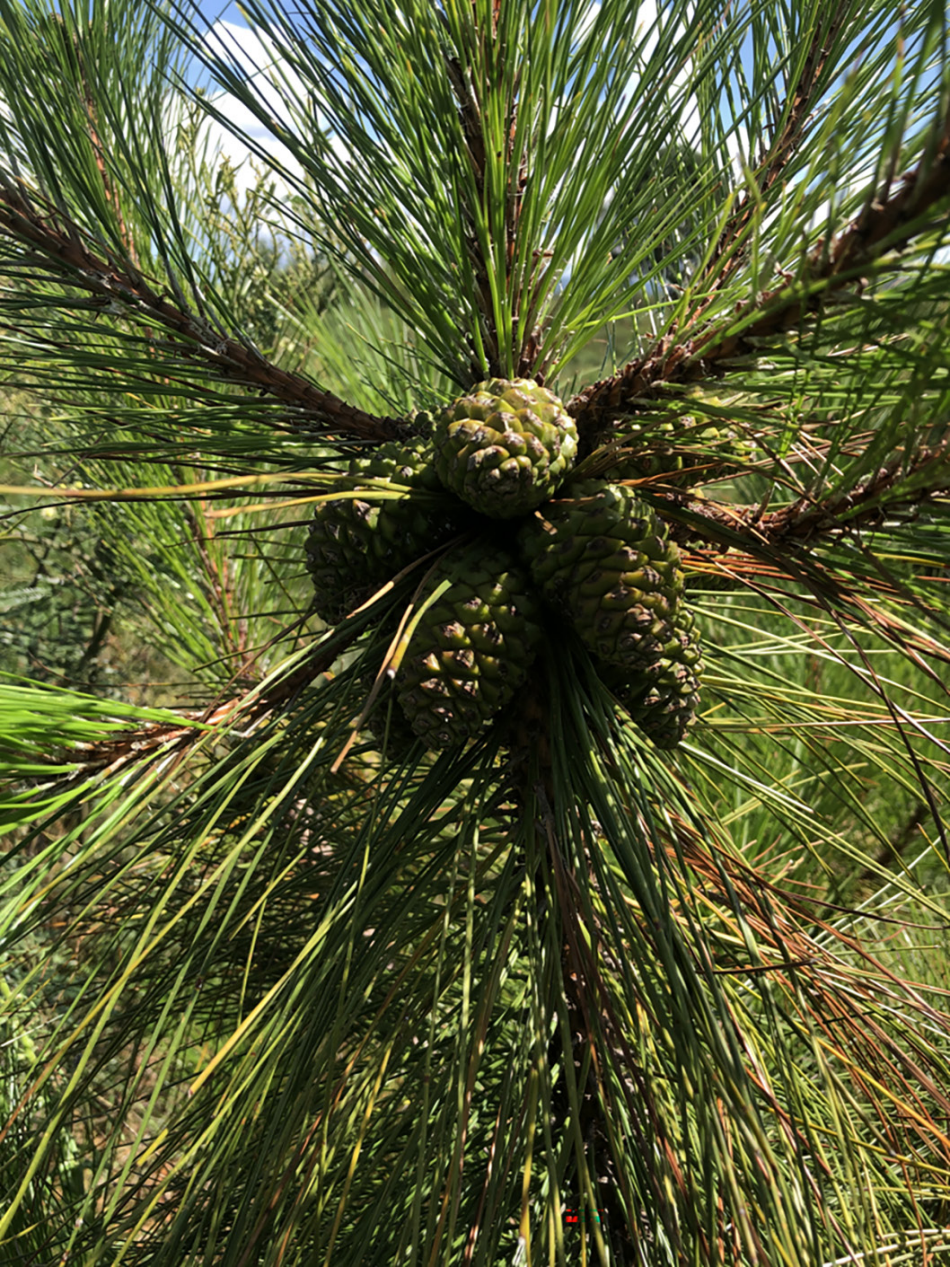
Mature cones of *Pinus yunnanensis* in its natural population.

**FIGURE 2:**
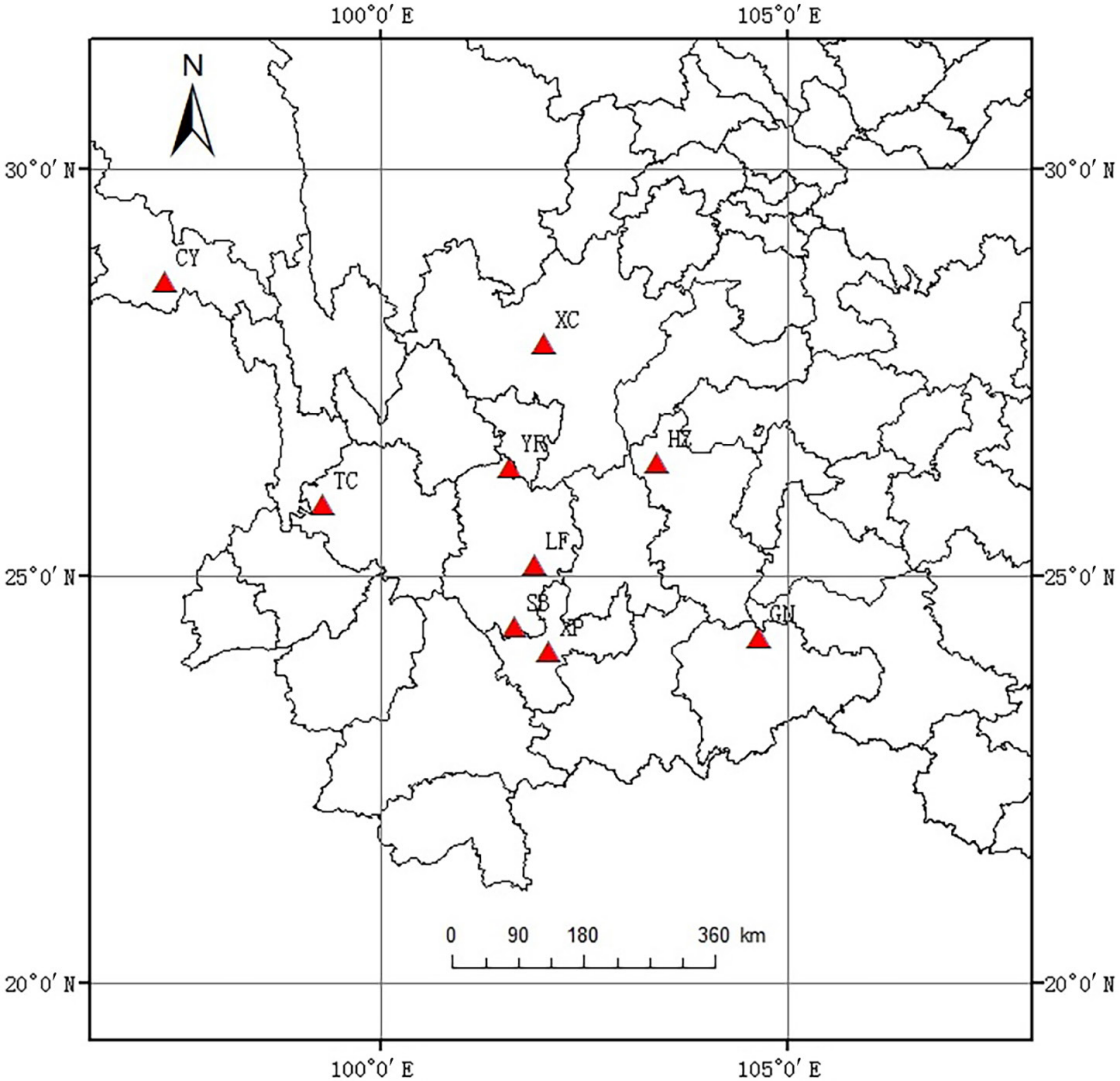
Geographical locations of sample cones collected from nine populations (red triangles) in the mountains of Southwest China.

*Pinus yunnanensis* is a wood tree of Pinaceae with the height of up to 30 m and the cones mature in October of the next year (Figure 1). It is naturally distributed in a variety of geological areas ranging from 23° to 30° N and 96° to 108° E and grows at elevation from 700 to 3000 m a.s.l. in Southwest China. The topography of *P. yunnanensis* is characterized by several large valley systems oriented in a north–south direction, perpendicular to the main Himalayan chain. These deep valleys combined with the surrounding high mountains are characterized by a variety of environment conditions (Wang et al., 2013). Controlled by the southwest and southeast monsoons, the climate in the distribution range of this species is characterized by dry and warm winters and humid and hot summers. The seasonal distribution of rain is uneven, and the dry season extends from November to April, accounting for 6%–17% of the annual precipitation, while the rainy season extends from the end of May to the end of September, and most of the precipitation is concentrated from June to August. There is a gradient of increasing precipitation, temperature, and humidity from north to south in the distribution range of *P. yunnanensis* (Chen et al., 2014). The distribution of the sample populations across the administrative districts and the bioclimatic zones was set in relation to the importance of *P. yunnanensis* cover within each of these geographical areas. The sampled natural *P. yunnanensis* populations were located in variable geographical sites belonging to nine counties in three provinces of Yunnan, Sichuan, and Tibet in Southwest China, covering the central and marginal distributions of this species and representing the current geographical distribution. The minimum distance between any two populations was approximately 30 km, and they were separated by a typical topographic feature such as a mountain ridge, forest, city, or river (Figure 2).

### 2.2 Data collection of tree, cone, and seed traits

Population selection and delimitation in each site were based on the requirements for the total absence of anthropogenic disturbance and forest pests and diseases, as well as covering a wide array of tree densities and canopy covers. About 30 open‐pollinated and healthy trees (older than 30 years) with ripe cones were selected for each population. The selected trees were located minimum 100 m apart from each other to minimize the chance of sampling the same genetic family. Tree height (*h*) and diameter at breast height (*d*) were measured at the site, and a minimum of 20 closed and mature cones with no signs of the presence of insects or diseases were collected from each tree by climbing and using an extendable pole pruner. Cones stored and transported in separate nylon mesh bags and identified by sample tree. In total, we sampled 2671 cones from 269 trees from nine populations and measured 14 traits of cone and seed in this study.

Ten ripe cones were selected at random from the 20 cones (or more) harvested from each tree, for measurements. Cone length (cl) and width (cw) of the widest part of each cone were recorded first. These cones were then allowed to be dried until the scales released completely, and the cone weight (cwe) was recorded. All scales on each cone were counted to obtain the number of total scales (csn), and every seed was extracted; seeds that did not fall out of the cones were removed with tweezers. Ten scales and seeds with wings for each cone were selected to measure seed length (sl), seed width (sw), seed wing length (ssl), seed wing width (ssw), seed scale length (scl), and seed scale width (scw).

The total seeds obtained from each cone were then floated in 100% ethanol to separate the filled ones from empty seeds and then rinsed with distilled water, air‐dried, and dry‐stored in paper bags at room where the temperature and relative humidity were maintained at 21°C and 15%, respectively. Moisture was drawn out of the seeds until water content was the same as that in the air. After drying, we counted and recorded the total number of filled seeds per cone (sn) and weighed them to the nearest 0.1 mg. The seed weight (sgw) was then estimated by dividing the total seed weight by the number of filled seeds. The scales in a pine cone can be divided into fertile and infertile scales, and each scale contains two ovules at its base. Thus, the number of aborted seeds (san; e.g., aborted ovules or empty seeds) for each cone was calculated by subtracting the number of filled seeds from twice the number of cone scales. We approximated an index of the seed abortion rate (sar) as (2 × scale number − filled seeds number)/(2 × scale number) (Wyse & Hulme, 2021).

### 2.3 Climate variables obtained

For each population, we quantified the mean climatic variables for a 48‐year time period (1970–2017) using ClimateAP ver.2.21 (Wang et al., 2017), which is a standalone software application that extracts and downscales gridded (4 × 4 km) monthly climate data for a given year or time period from Parameter‐elevation Regressions on Independent Slopes Model (PRISM) and WorldClim to scale‐free point locales. It also calculates monthly, seasonal, and annual climate variables. The downscaling is achieved through a combination of bilinear interpolation and dynamic local elevation adjustment. We focused on the hydrothermal climate indexes which are closely related to plant growth and development, such as mean annual temperature (MAT), mean warmest month temperature (MWMT), mean coldest month temperature (MCMT), growing degree‐days above 5°C (DD5), mean annual precipitation (MAP), Hargreaves climatic moisture deficit (CMD), and Hargreaves reference evaporation (Eref) rather than an exhaustive analysis of all variables. We also performed backward selection using the “ordistep” function in the “vegan” package in R (version 3.6.3; R Development Core Team, 2020), to eliminate highly collinear variables (MWMT and MCMT), and finally retained five climate variables (MAT, DD5, MAP, CMD, and Eref). A general geographical location and climatic characteristics of the populations are given in Table 1 and Table S1.

**TABLE 1:** Geographical location and climatic characteristics of nine *Pinus yunnanensis* populations sampled in the mountains of Southwest China.

Population	Geographical variables			Climatic variables (mean ± SD)				
	Latitude (°)	Longitude (°)	Elevation (m)	MAT (°C)	MAP (mm)	DD5	Eref	CMD
Yongren (YR)	26.34	101.60	2055	14.61 ± 0.39	1087 ± 127	3511 ± 134	1239 ± 24	554.5 ± 56.3
Lufeng (LF)	25.13	101.90	1925	16.19 ± 0.39	919 ± 114	4066 ± 137	1306 ± 25	574.6 ± 68.7
Huize (HZ)	26.40	103.40	2320	12.10 ± 0.38	836 ± 107	2676 ± 113	1096 ± 23	429.6 ± 64.7
Xinping (XP)	24.07	102.07	1600	17.81 ± 0.41	876 ± 106	4631 ± 138	1329 ± 26	560.1 ± 78.6
Chayu (CY)	28.62	97.35	2050	13.59 ± 0.41	741 ± 87	3195 ± 121	1235 ± 27	544.8 ± 88.1
Shuangbai (SB)	24.37	101.65	1655	20.45 ± 0.40	875 ± 103	5580 ± 142	1484 ± 29	692.9 ± 85.9
Xichang (XC)	27.87	102.01	2610	10.39 ± 0.39	1328 ± 133	2199 ± 97	1066 ± 24	339.6 ± 53.5
Tengchong (TC)	25.87	99.29	2607	11.88 ± 0.43	704 ± 73	2601 ± 128	1146 ± 22	522.3 ± 62.0
Guangnan (GN)	24.24	104.66	1780	16.24 ± 0.38	1201 ± 150	4087 ± 131	1244 ± 22	362.3 ± 75.1

*Note*: Mean annual temperature (MAT), mean annual precipitation (MAP), growing degree‐days above 5°C (DD5), Hargreaves reference evaporation (Eref), and Hargreaves climatic moisture deficit (CMD) for a 48‐year time period (1970–2017) were obtained using ClimateAP ver.2.21 (Wang et al., 2017).

Abbreviation: SD, standard deviation.

### 2.4 Statistical analyses

All statistical analyses were conducted in the R environment. Means, standard deviations, and coefficients of variation (CV) for each trait in each population were calculated. Mean values and CV for each trait within each population were standardized by Z‐score scaling and described by heat map using the “pheatmap” package in R. Unweighted group average method (UPGMA) clustering was also performed for the nine sampled populations by selecting “average” method in the “pheatmap” package. Nested variance analysis was performed to analyze the significance of differences and quantify the partitioning of variation in 14 phenotypic traits among and within populations using “aov” and “varcomp” functions, respectively. Multiple comparisons and significance difference test between populations were determined with a least significant difference (LSD) multiple range test. The phenotypic differentiation coefficient was calculated according to the proportion of the variance among populations to the total variance among and within population, which indicates the extent of the contribution of phenotypic variation among populations (Gandour et al., 2007). Pearson coefficients were performed by heat map using “ggcorrplot” and “psych” packages to analyze the correlation between CV and climatic and geographical variables, including latitude, longitude, elevation, MAP, MAT, DD5, CMD, and Eref. Meanwhile, linear regression was performed using the “lm” function to explore the response of CV of seed weight and number of filled seeds to the important geographical and climatic variables.

We determined the relative contribution of climatic, geographical, and tree size variables on cone and seed phenotypic datasets composed of multiple variables, with redundancy analyses (RDA) performed with the rda function in the “vegan” R package (version 3.6.3). RDA accounts for multiple response variables allowing to determine the effect of the multivariable on each individual phenotypic trait and in all traits as a whole. All data were homogenized by “Hellinger” transformation, and phenotypic databases were analyzed using multivariate linear regressions to produce multiple matrices of fitted values. We retained two main loads for significant eigenvalues (*p* < .05) and used them in subsequent analyses. Significance for explanatory variables was evaluated using Monte Carlo permutation (random simulation 999 times) by the envfit function in the “vegan” package, and the adjusted coefficient of determination (*r* ^2^; Peres‐Neto et al., 2006) was used to identify the importance of the explanatory variables. The “Varpart” function in “Vegan” package was used to conduct variance partitioning analysis to quantify the independent and joint ability of each set of explanatory variables to predict the variations in cone and seed phenotypic traits and to measure the importance and joint interactive effects of the variables together. Importantly, this also enabled us to quantify the amount of phenotypic variation not explained by environmental variation in our dataset.

## 3 RESULTS

### 3.1 Phenotypic trait and variation among and within populations

**FIGURE 3:**
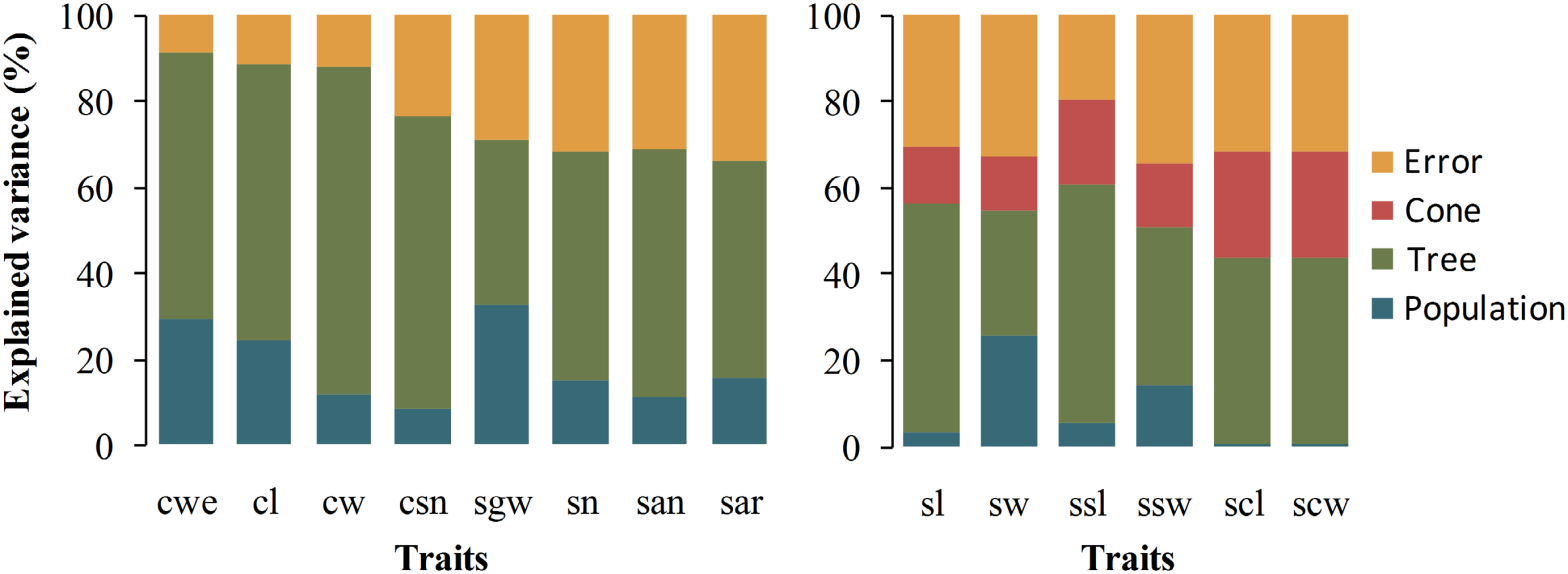
Variance components of phenotypic traits among and within populations for nine *Pinus yunnanensis* populations in the mountains of Southwest China. See Table 2 for abbreviations of phenotypic traits.

**FIGURE 4:**
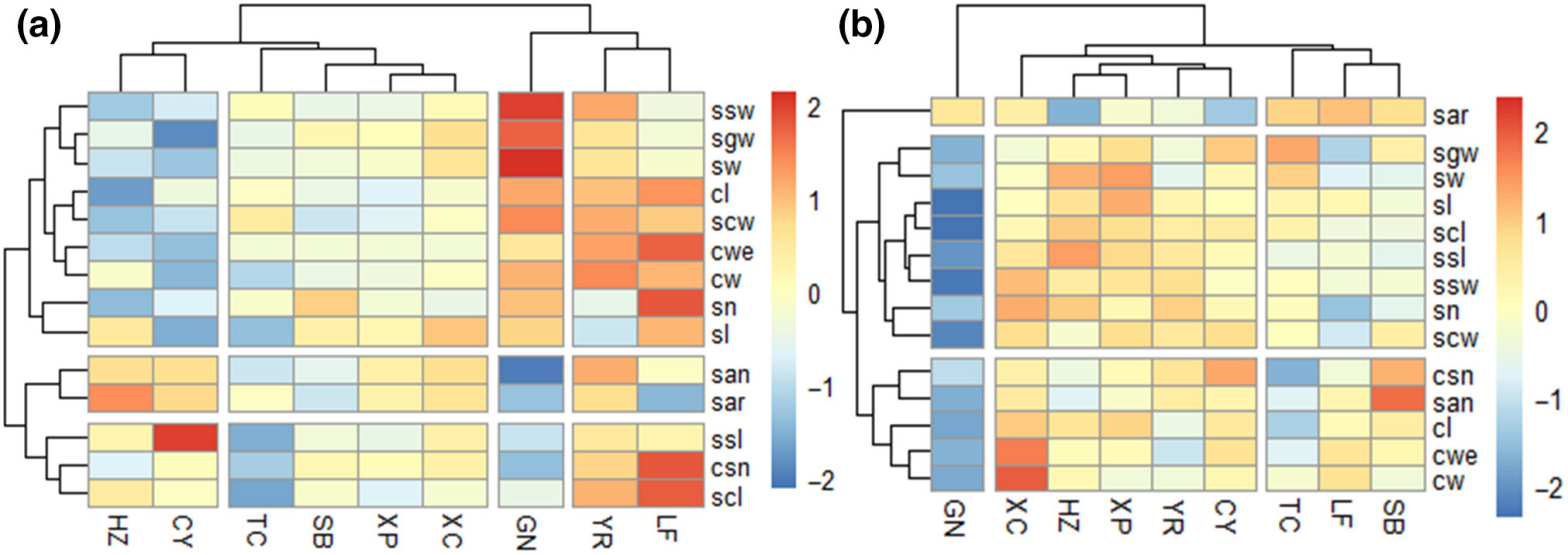
Standardized mean trait values (a) and variation coefficients of phenotypic traits (b) in different natural populations of *Pinus yunnanensis* in the mountains of Southwest China. The “pheatmap” package was used to describe the variations in the spatial pattern. Mean trait values and variation coefficients were standardized by Z‐score scaling. Unweighted group average method (UPGMA) cluster was performed based on the standardized data. See Table 2 for abbreviations of phenotypic traits.

Overall mean values, minimum and maximum values, and standard deviations (SD) showed that all cone and seed traits ranged widely across all populations (Table 2) and varied significantly both among and within populations (*p* < .001; Table 3 and Table S2). The average proportions of variance of the 14 phenotypic traits among populations and among individuals within population accounted for 14.17% and 52.15% of the total variation, respectively (Figure 3). The variance component within individual for seed length (sl), seed width (sw), seed wing length (ssl), seed wing width (ssw), seed scale length (scl), and seed scale width (scw) ranged from 12.79% to 24.85%. Phenotypic differentiation coefficients of all phenotypes among populations were less than 50% and the average value of the phenotypic differentiation coefficient of 14 traits was 18.92% (Table S3), which suggested that the contribution of interpopulation variation of *P. yunnanensis* was as low as 18.92% whereas the intrapopulation contribution was 81.08%. As interpopulation phenotypic variation was much lower than intrapopulation phenotypic variation, intrapopulation phenotypic variation of *P. yunnanensis* was the main source of cone and seed phenotypic variation. In addition, traits with the larger coefficients of differentiation were seed weight (45.68%), seed width (37.90%), and cone weight (32.39%), while the smallest were seed scale length (0.51%) and seed scale width (0.51%), indicating that the seed weight, seed width, and cone weight were more differentiated among populations than the other traits. For CV of cone and seed traits, the average CV was 16.73%, ranging from 9.44% to 51.24% across all populations. The smaller CV was observed in seed and cone size, that is, seed length (9.63%) and width (9.60%), cone length (12.10%) and width (9.44%), while the largest CV is the number of filled seeds per cone (51.24%), followed by cone weight (30.62%) and seed weight (20.27%; Figure 4b and Table S4).

**TABLE 2:** Sample cone and seed traits determined in nine *Pinus yunnanensis* populations in the mountains of Southwest China.

Trait	Description	Mean	Max	Min	SD
cwe	Cone weight (g)	31.32	82.11	12.67	9.59
cl	Cone length (mm)	73.44	101.49	48.11	8.88
cw	Cone width (mm)	39.27	54.41	29.41	3.71
csn	Number of total scales per cone (No.)	136	201	79	15
sgw	Mean seed weight per cone (mg)	18.917	32.845	7.582	3.835
sn	Number of filled seeds per cone (No.)	52	147	2	27
san	Number of aborted seeds per cone (No.)	219	387	119	5.634
sar	Seed abortion rate	0.808	0.993	0.512	0.093
sl	Seed length (mm)	5.71	8.26	3.11	0.55
sw	Seed width (mm)	3.45	5.44	2.00	0.33
ssl	Seed wing length (mm)	20.28	31.01	12.98	2.46
ssw	Seed wing width (mm)	6.64	11.02	3.17	0.83
scl	Seed scale length (mm)	22.49	35.07	12.23	3.35
scw	Seed scale width (mm)	13.47	21.11	4.84	1.79

Abbreviation: SD, standard deviation.

**TABLE 3:** Nested variance analysis of traits in cones and seeds among and within *Pinus yunnanensis* populations.

Trait	Mean square				*F* value		
	Among populations	Within population	Within individual	Residual	Among populations	Within population	Within individual
cwe	8933.32	591.16	NE	8.352	107.46***	71.38***	NE
cl	6385.16	529.28	NE	9.174	689.95***	57.11***	NE
cw	608.20	106.83	NE	1.691	356.95***	62.68***	NE
csn	7669.19	1655.37	NE	54.829	138.22***	29.82***	NE
sgw	1513.37	635.10	NE	4.420	341.64***	14.34***	NE
sn	37,035.07	4080.75	NE	233.737	158.09***	17.42***	NE
san	45,427.75	7089.41	NE	367.281	123.79***	19.32***	NE
sar	0.45	0.05	NE	0.003	148.53***	15.77***	NE
sl	12.34	5.05	0.48	0.093	132.78***	54.34***	5.11***
sw	15.71	1.23	0.19	0.040	388.47***	30.38***	4.79***
ssl	378.90	108.20	12.80	1.222	313.11***	89.39***	10.55***
ssw	63.36	9.04	1.29	0.261	248.38***	35.45***	5.04***
scl	207.77	167.59	30.69	3.527	58.81***	47.44***	8.69***
scw	273.41	35.75	6.63	1.369	198.90***	26.01***	4.88***

*Note*: NE indicates effects not introduced into the model; *** means the difference is significant at .001 level; see Table 2 for abbreviations of phenotypic traits.

### 3.2 Clustering analysis of phenotypic trait and variation

Based on the 14 phenotypic traits, nine populations were divided into four clusters (Figure 4a). HZ and CY populations were clustered together, which characterized lower values for most of the cone and seed traits but higher number of aborted seeds and seed abortion rate. GN population was clustered alone, showing higher values for most of the cone and seed traits but the lower number of aborted seeds and seed abortion rate. Based on the CV of 14 traits, nine populations were divided into three clusters (Figure 4b). The first cluster was GN population alone, showing the smallest CV of most traits. The second cluster included XC, HZ, XP, YR, and CY populations, characterized larger CV of most traits but smaller CV of seed abortion rate. The third cluster was composed of TC, LF, and SB populations, with characteristics of the largest CV of seed abortion rate. Combined with the geographical coordinates of each population sampling point (Figure 2), it was found that cone and seed phenotypic trait and variation were not clustered strictly according to geographical distance.

### 3.3 Influence of environmental factors on cone and seed phenotypic traits

**FIGURE 5:**
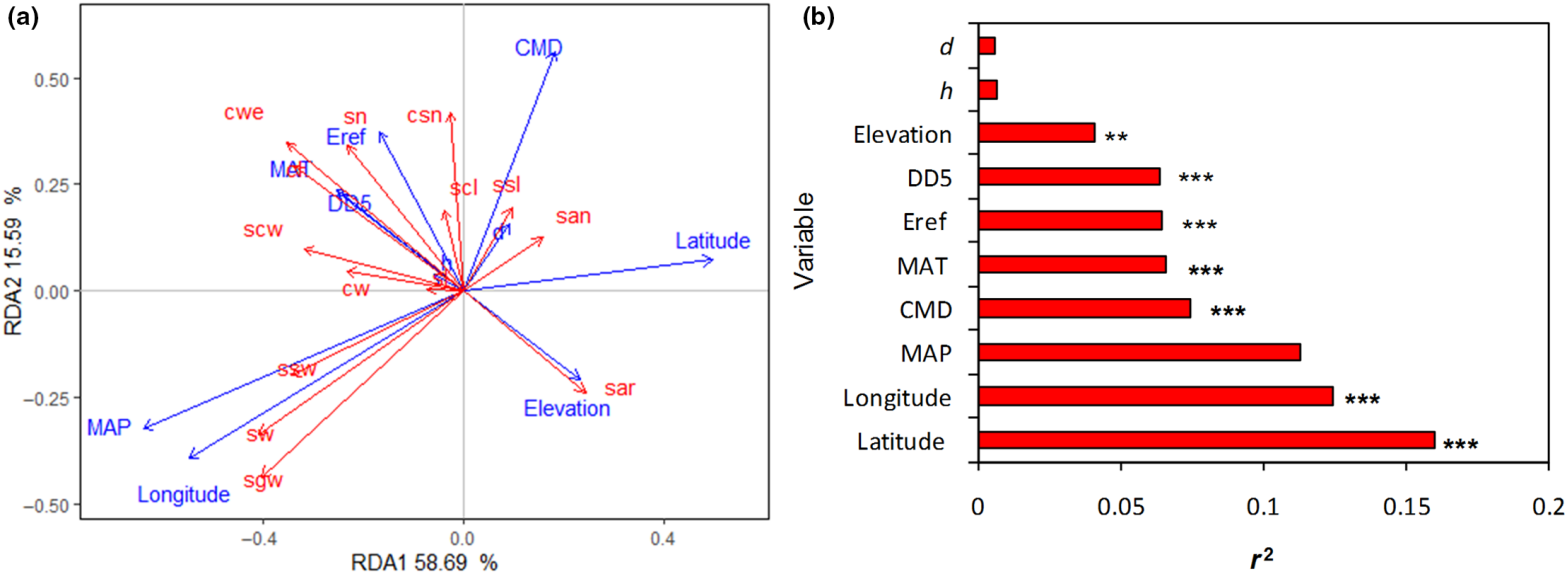
Relationships between cone and seed phenotypic traits of *Pinus yunnanensis* and climatic, geographical, and tree size variables based on redundancy analysis (a) and Monte Carlo permutation test (b). The lower *r* ^2^ value indicates the smaller impact of variable on cone and seed phenotypic traits; *** means the difference is significant at .001 level; ** means the difference is significant at .01 level. See Tables 1 and 2 for abbreviations of climatic variables and phenotypic traits, respectively.

Redundancy analysis (RDA) showed that the first two eigenvalues (RDA1 and RDA2) for this model explained up to 74.28% of the total variation. The first ordination axis (RDA1) mainly reflected the changes of phenotypic traits along the gradient of mean annual precipitation (MAP) and geographical variables such as longitude, latitude, and elevation, while the second ordination axis (RDA2) was mainly loaded by CMD, Eref, DD5, and MAT (Figure 5a). According to the length and direction of arrows, seed weight (sgw) and the width of seed and wing (sw and ssw) were positively correlated with MAP and longitude, while cone traits (i.e., cwe, cl, cw, scl, scw, and csn) and the number of filled seeds (sn) were mainly and positively related to MAT, Eref, and DD5. Latitude and elevation were mainly and positively related to seed abortion rate (sar) and number of aborted seeds (san), but negatively related to most of the other cone and seed traits. Monte Carlo permutation test showed that latitude, longitude, and MAP had larger important impact on phenotypic traits (*p* < .001), followed by CMD, MAT, Eref, DD5, and elevation (*p* < .01). However, both tree height and diameter did not significantly affect the variation in cone and seed traits (*p* > .05; Figure 5b).

**FIGURE 6:**
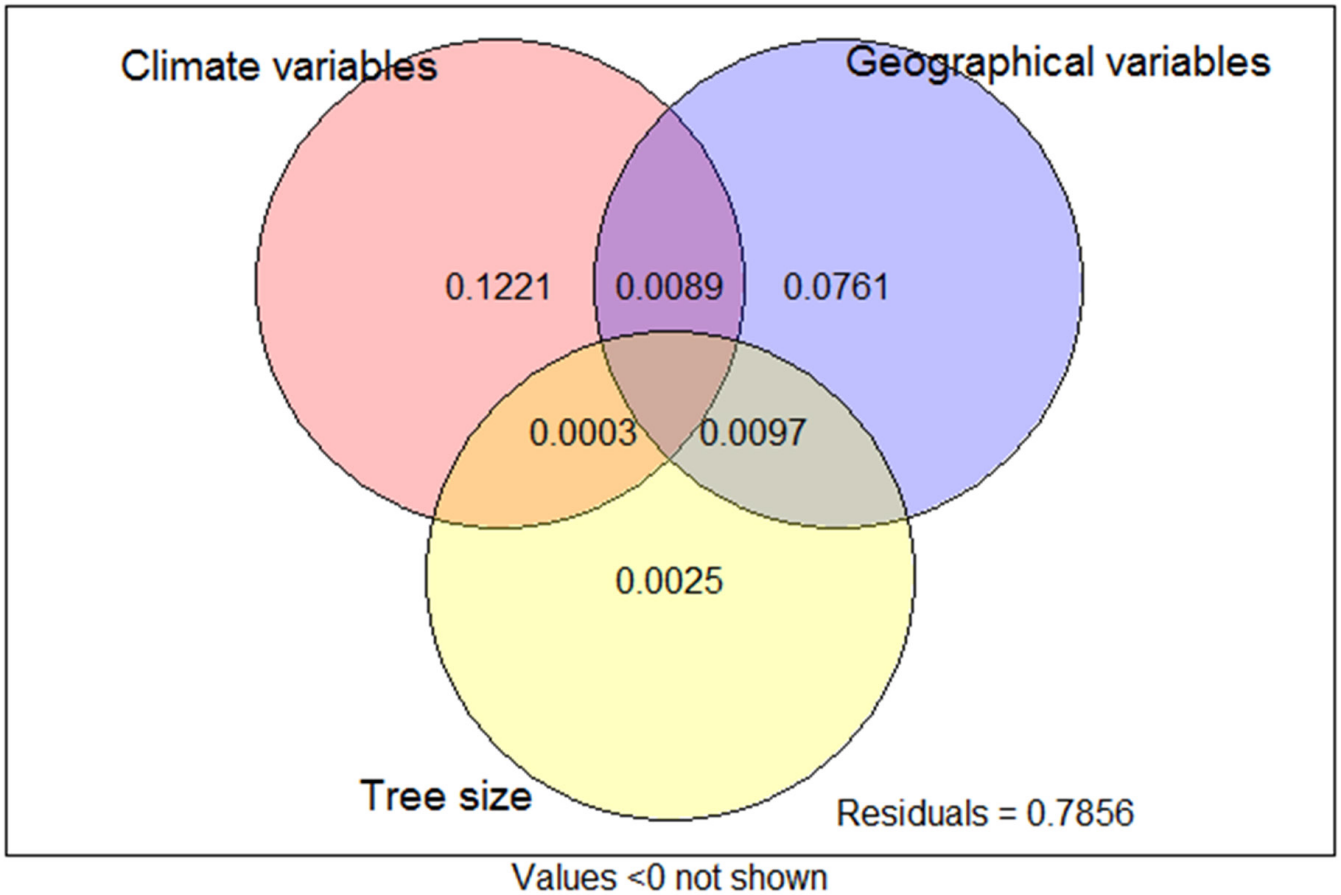
Variance partitioning diagram from partial redundancy analysis among (1) climatic, (2) geographical, and (3) tree size variable groups. The total explained variance in 14 morphological characteristics among all sampled individual cones is 21.44%, and the numbers in each compartment of the diagram indicate the amount of variance explained by the variable sets overlapping in that compartment.

Variance partitioning analysis (VPA) results showed that climatic, geographical, and tree size variables explained 21.44% of the total variation in phenotypic traits among sampled cones (Figure 6 and Table S5). Of the marginal effects of these groups of variables, climatic variables were the main contributor to variation in cone and seed phenotypic traits, accounting for 12.61% of the variance, followed by geographical variables (8.96%), whereas the contribution of tree size variables to the variation of phenotypic traits only accounted for 0.73%.

### 3.4 Correlations between coefficient of variation and climatic and geographical variables

**FIGURE 7:**
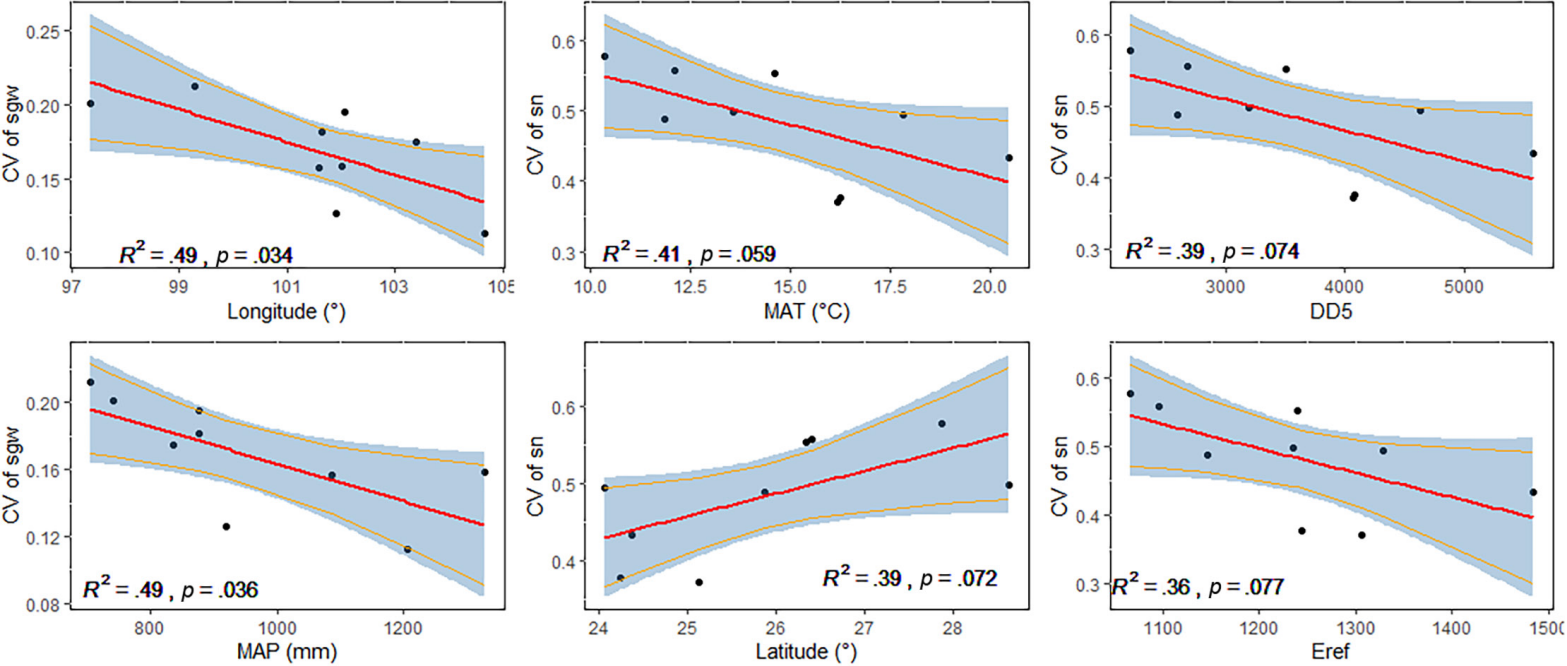
Relationships between the climatic and geographical variables and variation coefficients of seed weight (CV of sgw) and number of filled seeds (CV of sn) of the nine study populations. The slope (red line) and 95% confidence interval (blue area) are based on the linear model.

The CV for most of the traits associated with cone and seed size (i.e., cl, sl, sw, ssl, ssw, and scl) in different populations was nonsignificantly correlated with climatic and geographical variables (Figure S1). However, the CV of seed weight (CV of sgw) was significantly and negatively correlated with longitude (*R* ^2^ = .49, *p* = .034) and mean annual precipitation (*R* ^2^ = .49, *p* = .036). The CV of number of filled seeds (CV of sn) was marginally and positively related to latitude (*R* ^2^ = .39, *p* = .072) and elevation (*R* ^2^ = .34, *p* = .099), but negatively related to mean annual temperature (*R* ^2^ = .41, *p* = .059), DD5 (*R* ^2^ = .39, *p* = .074), and Eref (*R* ^2^ = .36, *p* = .077; Figure 7).

## 4 DISCUSSION

### 4.1 Variation patterns in cone and seed phenotypic traits

Several important patterns of reproductive variation in *P. yunnanensis* were detected. First, intrapopulation phenotypic variation was the main source of cone and seed phenotypic variation, as we found that phenotypic differentiation coefficients among populations of all cone and seed traits were less than 50% and the average value of the phenotypic differentiation coefficient of 14 traits was only 18.92% (Figure 3). This result was consistent with previous studies based on genetic diversity analysis using DNA markers and allozymes that indicated the genetic differentiation coefficient of *P. yunnanensis* among populations was 7% (Xu et al., 2016) and 13.4% (Gst = 0.134; Yu et al., 2000). Such a variation pattern was similar to other *Pinus* species such as *P. tabulaeformis* (Ji et al., 2011) and *P. kesiya* var. *langbianensis* (Li et al., 2013) and are common in coniferous plants characterized by outcrossing with large distribution areas. The breeding system may be the main source of variation within the population. High outcrossing rates in conifers maintain high intrapopulation genetic diversity, and wind‐dispersed pollen usually results in high levels of gene flow (Rubio‐Moraga et al., 2012). *P. yunnanensis* has high outcrossing rates and a general intolerance of selfing. This cross‐pollinated plant with winged seeds and air sacs in pollen can be dispersed at a long distance, resulting in high levels of gene flow between populations and weakening genetic differentiation between populations. Additionally, microecological environments acting on the plasticity of individuals, both light availability and soil conditions could also be important for trait variation within population (Lemke et al., 2015).

Second, we found that cone and seed phenotypic trait and variation were not clustered strictly according to geographical distance, and the larger CV for the populations (e.g., XC, HZ, XP, and CY) lying at the edge of northern and southern regions was higher than that in the mid‐regions (e.g., TC, LF, and SB; Figure 4b). This geographical variation pattern implied that there is discontinuity in the variation of cone and seed traits and may have resulted from two sets of factors, one of which consists of the heterogeneous ecological factors that may lead to the significant differentiation of reproductive traits among populations of coniferous plants (Liu & El‐Kassaby, 2020; Marcysiak, 2004). Southwest China is characterized by a number of large valley systems that create widely differing microecological environments among locations and elevations (Wang et al., 2013). Phenotypic plasticity or long‐term adaptation owing to heterogeneous ecological environments may maintain high variability in reproductive traits (Cochrane et al., 2015) and create stronger and more discrete phenotypic differentiation than isolation by distance alone (Wang et al., 2013). On the contrary, also genetic causes may be responsible for the observed patterns, as phenotypic variation may not only be caused by differences in the environment but also by differences in the underlying genotypic variation (Arenas et al., 2021). A higher trait variation may thus also be the result of a greater genetic variability (Cushman, 2014; Sork et al., 2013) that may vary across the latitudinal gradient. *P. yunnanensis* is adjacent to *P. kesiya* var. *langbianensis* to southwest and *P. densata* to northwest (Jin & Peng, 2004). The larger reproductive trait variation for the populations in northern and southern regions may also be affected by gene flow from *P. densata* and *P. kesiya* var. *langbianensis*, respectively, which are capable of introgressive hybridization with *P. yunnanensis* (Mao et al., 2009; Wang et al., 2013; Yu et al., 2000). However, to disentangle the underlying genetic and phenotypic components of the detected trait variation, additional reciprocal transplant experiments or multiple common garden studies are needed (Cushman, 2014).

### 4.2 Effects of environmental factors on phenotypic traits

Phenotypic traits of species vary along environmental gradients in a predictive way and thus determine species distributions across broad environmental gradients due to environmental filters (Lemke et al., 2015; Saatkamp et al., 2019; Wu et al., 2018). As predicted, we found the various patterns of the correlations of cone and seed phenotypic traits and the environmental factors. Seed weight was positively corrected with MAP and longitude, while cone traits (i.e., cwe, cl, cw, scl, scw, and csn) and filled seed number were mainly and positively related to MAT, Eref, and DD5 (Figure 5). This result indicated that cone and seed weight and seed number increased southeastward with precipitation and temperature rising, which are consistent with previous studies that have examined relationships between cone or seed traits and climatic variables (Cochrane et al., 2016; Leal‐Sáenz et al., 2020; Middleton et al., 2021; Murray et al., 2004), but in contrast to earlier work in other tree species by Baker (1972) and Liu et al. (2013) who found that seeds were larger in drier sites. These differences suggest that generalizations are difficult because individual species vary in their reproductive responses to climate and different outcomes were affected by the species and the extent of sampling (Moles et al., 2007; Soper‐Gorden et al., 2016). We detected that latitude and elevation were positively related to seed abortion rate and number of aborted seeds, but negatively related to most of the other cone and seed traits (e.g., cone weight and seed weight and seed number). The decline in cone and seed weight and the increase in seed abortion rate and number of aborted seeds with elevation and latitude increasing may be due to environmentally induced plastic responses to a decline in resource availability (Moles et al., 2007). Low temperature atmospheric pressure at higher elevation and latitude may decrease photosynthetic rates, and shorter growing seasons may reduce the time for seed development and seed provisioning, thereby reducing mature seed mass and filled seed number and enhancing seed abortion rate (Baker, 1972; Guo et al., 2010; Wang et al., 2014). The production of smaller seeds at high latitude and altitude may also evolve by natural selection if the growing season is not sufficiently long for large‐seeded genotypes to produce mature seeds (Guo et al., 2010; Murray et al., 2004).

Plant size is an important determinant of the amount of energy available for reproduction and seed development (Guo et al., 2010; Wang et al., 2014). Indeed, positive relationships between seed mass and plant size have been reported in many previous studies (Venable & Rees, 2009; Wang et al., 2014). These positive relationships could be contributed to the mechanical constraints hypothesis predicts that the fragile branches of small plants should only be able to bear relatively small seeds, whereas large plants should be robust enough to bear either large or small seeds (Grubb et al., 2005). Additionally, factors such as the intensity of competition for light and the height requirements needed for the successful dispersal of large seeds may contribute to a positive relationship between seed mass and plant size (Guo et al., 2010). In the present study, we detected no significant relationship between tree size (e.g., tree height and diameter) and cone or seed traits (Figure 5b). This result could be attributed to the samplings characterized by a single tree species with an older age than 30 years. So, there may not be sufficient variation in plant size to detect a strong positive correlation with cone and seed traits. This suggests that different mechanisms may operate at different ecological levels (e.g., within vs. across species), as has been found previously, cone or seed traits were significantly positively correlated with plant height among populations across species, but not within species (Guo et al., 2010; Leal‐Sáenz et al., 2020; Wang et al., 2014).

In terms of environmental factors in the present study, we found that climatic, geographical, and tree size variables explained 21.44% of the total variation in phenotypic traits (Figure 6), which suggested that there may be other important environmental variables that significantly affect the variation in cone and seed traits, such as soils (Arenas et al., 2021), topography, and vegetation (Leal‐Sáenz et al., 2020). The independent explanatory power of climate, geographical, and tree size variable sets reported here, however, is useful in showing the importance of environmental factors in promoting and maintaining phenotypic variation across a complex landscape, and climatic variables are more important than geographical and tree size variables in their relationships to cone and seed traits.

### 4.3 Effects of geo‐climate factors on the variation in phenotypic traits

Most importantly, apart from trait performance, we found that geo‐climate factors also affected intrapopulation phenotypic variation of several important traits (Figure 7). To explain the relationship between trait variation within population and climate, two opposing hypotheses have been proposed. The stress‐reduced variability hypothesis states that trait variation decreases with extreme abiotic conditions that generate stress, resulting in a decreased trait variation in cold and dry climates and in nutrient‐poor sites (Hulshof et al., 2013; Lemke et al., 2012, 2015). Extreme abiotic conditions have the potential to act as an environmental filter and/or strong selective agent, causing trait convergence within species and thus reducing phenotypic variation (Caruso et al., 2017; Kuppler et al., 2020). The stress‐induced variability hypothesis, however, posits that unfavorable conditions trigger enhanced expression of phenotypic variability in traits, thus resulting in increased trait variation (Helsen et al., 2017). In stressful conditions, phenotypic variation may increase owing to developmental instability and higher rates of recombination and mutation, in addition to competition avoidance when resources become less abundant (Hoffmann & Merilä, 1999; Siefert et al., 2015). Our results showed that CV of seed weight was significantly and negatively correlated with longitude and mean annual precipitation, while CV of the number of filled seeds was marginally negatively related to mean annual temperature. These findings may reflect the high variability of seed traits in the drier and colder climate and are similar to the previous study that found strong negative correlations between nuclear DNA content and precipitation and temperature in the genus *Pinus*, suggesting that higher levels of intrapopulation seed trait variation may be adapted responses to the habitats of these species (Wakamiya et al., 1993). Our results supported the stress‐induced variability hypothesis. This is also in agreement with previous studies focusing on fewer species, which found induced variability in seed mass at low levels of precipitation (Helsen et al., 2017). Several mechanisms might potentially explain the increasing trait variation in harsher climate. First, it can result from increased genetic variation in stressful conditions (Hoffmann & Merilä, 1999; Wakamiya et al., 1993). Second, increased trait variation within population might be attributable to reduced canalization in development (Valladares et al., 2014) and thus increasing development instability. Third, large trait variation in harsher climate might result from local variation in microclimatic conditions, because water availability can be proportionally more variable across microsites when precipitation is low, leading to greater plasticity or, in certain conditions, local genetic differentiation (Kuppler et al., 2020). In general, we found that the intrapopulation phenotypic variation of traits relevant to reproductive fitness was correlated with specific climate conditions as described above. This relationship occurs for seed weight and seed number, but not necessarily in all reproductive traits. Our results support the recent findings of inconsistent effects of climate on intrapopulation variation of traits (Henn et al., 2018; Kuppler et al., 2020; Lemke et al., 2015) and suggest that the phenotypic variations in seed weight and seed number are more sensitive to climatic stress than the other traits.

## 5 CONCLUSION

In summary, our results demonstrate that natural populations of *P. yunnanensis* in the mountains of Southwest China exhibit significant variations in cone and seed traits both among and within populations and the intrapopulation variation accounted for a majority of the total variation. Additionally, we found that both phenotypic trait and variation were not clustered strictly according to geographical distance and the larger CV for the populations lying at the edge of northern and southern regions. Climatic variables are more important than geographical and tree size variables in their relationships to cone and seed traits, and populations in more humid and warmer climate expressed greater cone and seed weight and seed number. Most importantly, intrapopulation variation of seed mass and seed number tends to be increased with climatic stress (drier or colder climate), due to either a higher phenotypic plasticity or a larger genetic variation, which supported the stress‐induced variability hypotheses, and might foster plant‐population persistence in stressful conditions. Our study illustrates that intraspecific trait variation should be considered when examining plant species response to changing climate; however, additional reciprocal transplant experiments or multiple common garden studies are required to disentangle the phenotypic and genotypic components of the detected trait variation.

## AUTHOR CONTRIBUTIONS

**Chengjie Gao:** Formal analysis (lead); funding acquisition (equal); investigation (equal); writing – original draft (lead). **Fangyan Liu:** Formal analysis (equal); investigation (equal). **Yingchun Miao:** Data curation (equal); investigation (equal). **Jin Li:** Investigation (equal); software (equal). **Zirui Liu:** Formal analysis (equal); investigation (equal). **Kai Cui:** Funding acquisition (equal); project administration (lead); supervision (equal); writing – review and editing (equal).

## CONFLICT OF INTEREST STATEMENT

The authors declare no conflict of interest.

## Supporting information


Data S1:

## Data Availability

The data supporting the results are available in a public repository at: https://datadryad.org/stash/share/l‐2QpIYfj4DuPa75YzYG8u3d3_2gu034wk4MbRoh3v4.
