# Supplementary material for: Effects of geo‐climate factors on phenotypic variation in cone and seed traits of Pinus yunnanensis
Source: Ecol Evol. 2023 Sep 28;13(10):e10568. doi: 10.1002/ece3.10568 (PMC10534196; doi:10.1002/ece3.10568)
Supplement: Supplementary file 1 — Data S1: [file ECE3-13-e10568-s001.docx]

Supplementary Material

**Supplementary Table 1 |** Sample cone and seed attributes of geographical location and climatic characteristics determined in nine *Pinus* *yunnanensis* populations in the mountains of Southwest China. Climatic variables for a 48-year time period (1970-2017) were obtained using ClimateAP ver.2.21 (Wang et al., 2017). SD = standard deviation.

| Variable | Description | Min | Max | Mean | SD |
| --- | --- | --- | --- | --- | --- |
| Latitude | Latitude (°) | 24.07 | 28.62 | 25.88 | 1.61 |
| Longitude | Longitude (°) | 97.35 | 104.66 | 101.55 | 2.13 |
| Elevation | Elevation (m) | 1600 | 2610 | 2067 | 378 |
| MAT | Mean annual temperature (°C) | 10.4 | 20.5 | 14.8 | 3.2 |
| DD5 | Degree-days above 5°C, growing degree-days | 2199 | 5580 | 3616 | 1085 |
| MAP | Mean annual precipitation (mm) | 703.7 | 1327.8 | 952.4 | 211.0 |
| CMD | Hargreaves climatic moisture deficit | 339.6 | 692.9 | 509.0 | 112.2 |
| Eref | Hargreaves reference evaporation | 1065.5 | 1484.2 | 1238.2 | 128.4 |

**Supplementary Table 2 |** Means (SD) analysis of cone and seed traits in nine *Pinus* *yunnanensis* populations in the mountains of Southwest

| **Variable** | **Population** | | | | | | | | |
| --- | --- | --- | --- | --- | --- | --- | --- | --- | --- |
|  | **YR** | **LF** | **HZ** | **XP** | **CY** | **SB** | **XC** | **TC** | **GN** |
| cwe | 38.120 b | 40.969 a | 25.967 e | 29.883 d | 23.663 f | 29.761 d | 29.921 d | 29.885 d | 34.228 c |
|  | (7.824) | (11.736) | (6.684) | (7.797) | (6.819) | (7.863) | (10.180) | (6.486) | (5.802) |
| cl | 77.97 b | 79.89 a | 65.56 f | 70.23 e | 71.61 d | 71.50 d | 72.63 cd | 73.33 c | 79.08 ab |
|  | (7.38) | (8.57) | (7.66) | (8.57) | (8.28) | (8.12) | (8.99) | (5.84) | (5.46) |
| cw | 41.34 a | 40.79 a | 39.08 bc | 38.78 bc | 37.20 d | 38.63 c | 39.22 b | 37.72 d | 40.86 a |
|  | (3.32) | (4.05) | (3.52) | (3.10) | (3.38) | (3.08) | (4.87) | (3.12) | (2.20) |
| csn | 140 a | 145 b | 132 d | 136 c | 136 c | 137 c | 137 c | 129 e | 129 e |
|  | (16) | (14) | (13) | (15) | (17) | (17) | (15) | (10) | (11) |
| sgw | 20.253 b | 18.344 d | 17.760 e | 18.966 c | 14.606 f | 19.365 c | 20.419 b | 17.849 de | 22.936 a |
|  | (3.177) | (2.315) | (3.101) | (3.702) | (2.939) | (3.510) | (3.228) | (3.786) | (2.585) |
| sn | 46 de | 72 a | 36 f | 49 cd | 45 e | 62 b | 47 cde | 51 c | 63 b |
|  | (26) | (27) | (20) | (24) | (22) | (27) | (27) | (25) | (24) |
| san | 233 a | 218 d | 228 b | 223 cd | 228 bc | 212 e | 228 bc | 208 e | 194 f |
|  |  |  |  |  |  |  |  |  |  |
|  | (35) | (32) | (30) | (32) | (34) | (36) | (35) | (28) | (23) |
| sar | 0.835 b | 0.751 f | 0.862 a | 0.819 c | 0.837 b | 0.775 e | 0.831 bc | 0.805 d | 0.756 f |
|  | (0.086) | (0.089) | (0.076) | (0.086) | (0.076) | (0.089) | (0.092) | (0.094) | (0.086) |
| sl | 5.61 c | 5.85 a | 5.78 b | 5.75 b | 5.53 d | 5.76 b | 5.84 a | 5.55 d | 5.82 ab |
|  | (0.53) | (0.55) | (0.58) | (0.62) | (0.50) | (0.50) | (0.53) | (0.52) | (0.34) |
| sw | 3.59 b | 3.45 cd | 3.32 g | 3.46 c | 3.24 f | 3.43 de | 3.59 b | 3.42 e | 3.89 a |
|  | (0.28) | (0.26) | (0.33) | (0.35) | (0.28) | (0.27) | (0.30) | (0.33) | (0.26) |
| ssl | 20.55 b | 20.40 b | 20.40 b | 19.88 cd | 21.60 a | 20.01 c | 20.45 b | 19.17 f | 19.60 d |
|  | (2.58) | (2.06) | (3.09) | (2.67) | (2.43) | (1.81) | (2.60) | (1.81) | (0.96) |
| ssw | 7.13 b | 6.58 d | 6.27 f | 6.55 d | 6.44 e | 6.54 d | 6.75 c | 6.74 c | 7.41 a |
|  | (0.91) | (0.72) | (0.78) | (0.83) | (0.73) | (0.72) | (0.92) | (0.77) | (0.54) |
| scl | 23.02 b | 23.36 a | 22.70 bc | 22.12 e | 22.44 cd | 22.39 cde | 22.36 de | 21.67 f | 22.23 cde |
|  | (3.51) | (3.07) | (3.74) | (3.55) | (3.44) | (2.94) | (3.25) | (3.21) | (1.86) |
| scw | 14.28 a | 14.11 b | 12.70 g | 13.15 e | 12.99 f | 13.01 ef | 13.50 d | 13.84 c | 14.46 a |
|  | (1.86) | (1.58) | (1.53) | (1.76) | (1.74) | (1.68) | (1.81) | (1.71) | (1.38) |

Means in the same row followed by the different letters are statistically different at a 0.05 significance level using the LSD post hoc test.

**Supplementary Table 3 |** Variance components and phenotypic traits differentiation coefficient among populations, among individuals and within individual for nine *Pinus yunnanensis* populations in the mountains of Southwest.

| **Variables** | **Explained variance (%)** | | | |  | **Phenotype differentiation coefficient (%)** |
| --- | --- | --- | --- | --- | --- | --- |
|  | **Among populations** | **Among individuals** | **Within individual** | **error** |  |  |
| cwe | 29.56 | 61.72 | - | 8.72 |  | 32.39 |
| cl | 24.46 | 64.16 | - | 11.38 |  | 27.60 |
| cw | 12.00 | 75.77 | - | 12.22 |  | 13.67 |
| csn | 8.63 | 67.92 | - | 23.45 |  | 11.27 |
| sgw | 32.55 | 38.71 | - | 28.74 |  | 45.68 |
| sn | 15.24 | 52.85 | - | 31.91 |  | 22.38 |
| san | 11.25 | 57.58 | - | 31.17 |  | 16.35 |
| sar | 15.38 | 50.64 | - | 33.98 |  | 23.29 |
| sl | 3.25 | 52.88 | 13.37 | 30.50 |  | 4.68 |
| sw | 25.58 | 29.12 | 12.79 | 32.51 |  | 37.90 |
| ssl | 5.53 | 55.15 | 19.57 | 19.75 |  | 6.89 |
| ssw | 14.25 | 36.78 | 14.62 | 34.35 |  | 21.71 |
| scl | 0.35 | 43.38 | 24.85 | 31.42 |  | 0.51 |
| scw | 0.35 | 43.38 | 24.85 | 31.42 |  | 0.51 |

.**Supplementary Table 4 |** Variation coefficients (%) of cone and seed traits in nine *Pinus* *yunnanensis* populations in the mountains of Southwest

| **Variable** | **Population** | | | | | | | | | **All populations** |
| --- | --- | --- | --- | --- | --- | --- | --- | --- | --- | --- |
|  | **YR** | **LF** | **HZ** | **XP** | **CY** | **SB** | **XC** | **TC** | **GN** |  |
| cwe | 20.53 | 28.65 | 25.74 | 26.09 | 28.82 | 26.42 | 34.02 | 21.70 | 16.95 | 30.62 |
| cl | 9.46 | 10.72 | 11.68 | 12.20 | 11.56 | 11.35 | 12.38 | 7.97 | 6.90 | 12.10 |
| cw | 8.04 | 9.93 | 9.00 | 7.99 | 9.08 | 7.96 | 12.41 | 8.28 | 5.37 | 9.44 |
| csn | 11.63 | 9.82 | 9.62 | 10.69 | 12.84 | 12.71 | 11.12 | 7.38 | 8.41 | 11.27 |
| sgw | 15.69 | 12.62 | 17.46 | 19.52 | 20.12 | 18.12 | 15.81 | 21.21 | 11.27 | 20.27 |
| sn | 55.24 | 37.09 | 55.72 | 49.43 | 49.72 | 43.30 | 57.77 | 48.82 | 37.66 | 51.24 |
| san | 15.09 | 14.81 | 13.36 | 14.15 | 14.82 | 17.11 | 15.16 | 13.37 | 11.91 | 15.52 |
| sar | 10.29 | 11.86 | 8.77 | 10.44 | 9.09 | 11.46 | 11.12 | 11.64 | 11.31 | 11.56 |
| sl | 9.43 | 9.33 | 10.03 | 10.77 | 9.08 | 8.61 | 9.06 | 9.43 | 5.82 | 9.63 |
| sw | 7.80 | 7.62 | 10.01 | 10.21 | 8.74 | 7.81 | 8.49 | 9.68 | 6.77 | 9.60 |
| ssl | 12.55 | 10.11 | 15.14 | 13.43 | 11.24 | 9.03 | 12.72 | 9.46 | 4.90 | 12.15 |
| ssw | 12.81 | 10.92 | 12.43 | 12.63 | 11.36 | 11.05 | 13.57 | 11.42 | 7.27 | 12.57 |
| scl | 15.27 | 13.13 | 16.45 | 16.06 | 15.31 | 13.12 | 14.53 | 14.83 | 8.37 | 14.87 |
| scw | 13.06 | 11.20 | 12.08 | 13.42 | 13.42 | 12.92 | 13.38 | 12.39 | 9.57 | 13.31 |
| Mean value | 15.49 | 14.13 | 16.25 | 16.22 | 16.09 | 15.07 | 17.25 | 14.83 | 10.89 | 16.73 |

**Supplementary Table 5** | Results of RDA analysis and variance partitioning.

| **Component** | **Description of Component** | **Variance Explained by Component** |
| --- | --- | --- |
| Marginal x1 | Climate Total | 0.1261 |
| Marginal x2 | Geographic- Total | 0.0896 |
| Marginal x3 | Tree size | 0.0073 |
| x1 | Climate Only | 0.1221 |
| x2 | Geographic Only | 0.0761 |
| x3 | Tree size Only | 0.0025 |
| x1x2 | Climate and Geographic Only | 0.0089 |
| x1x3 | Climate and Tree size Only | 0.0003 |
| x2x3 | Geographic and Tree size Only | 0.0097 |
| x1x2x3 | Climate, Geographic and Tree size | 0 |


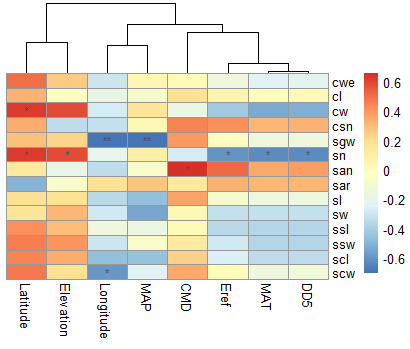


**Supplementary FIGURE 1|** Correlation coefficients (r values) between variation coefficients of phenotypic traits and climatic and geographical variables. ** means difference is significant at 0.05 level; * means difference is significant at 0.1 level.
